# Comparison of outcomes of monochorionic twin pregnancies conceived by assisted reproductive technology vs. spontaneous conceptions: A systematic review and meta-analysis

**DOI:** 10.3389/fped.2022.962190

**Published:** 2022-10-13

**Authors:** Minmin Wang, Jingjing Chai

**Affiliations:** Department of Obstetrics, The First People’s Hospital of Fuyang, Hangzhou, China

**Keywords:** twin pregnancies, monochorionic, neonatal outcomes, in-vitro fertilization, assisted reproductive technology

## Abstract

**Background:**

This review aimed to assess if monochorionic twin pregnancies conceived by assisted conception have worse maternal and neonatal outcomes as compared to those conceived naturally.

**Methods:**

Datasets of PubMed, ScienceDirect, CENTRAL, Embase, and Google Scholar were searched for studies comparing maternal and neonatal outcomes of monochorionic twin pregnancies conceived by assisted vs. spontaneous methods.

**Results:**

Eight studies comparing 337 assisted with 2,711 spontaneously conceived monochorionic twin pregnancies were included. Meta-analysis revealed that the mode of conception of monochorionic twin pregnancies had no impact on the risk of hypertensive disorders of pregnancy (HDP) (OR: 1.36 95% CI, 0.73, 2.54 *I*^2^ = 9% *p* = 0.03), twin-twin transfusion syndrome (TTTS) (OR: 0.83 95% CI, 0.52, 1.31 *I*^2^ = 0% *p* = 0.42), and very preterm delivery (OR: 1.18 95% CI, 0.74, 1.88 *I*^2^ = 41% *p* = 0.49). We noted no statistically significant difference in the mean birth weights (MD: −17.66 95% CI, −157.23, 121.91 *I*^2^ = 82% *p* = 0.80), risk of intra-uterine death (OR: 0.90 95% CI, 0.51, 1.60 *I*^2^ = 36% *p* = 0.73) and small for gestational age between the two groups (OR: 0.92 95% CI, 0.67, 1.26 *I*^2^ = 0% *p* = 0.59). There was an increased risk of caesarean sections (OR: 1.34 95% CI, 1.00, 1.80 *I*^2^ = 0% *p* = 0.05) and neonatal death with assisted conceptions as compared to spontaneous conceptions (OR: 2.35 95% CI, 1.11, 5.01 *I*^2^ = 37% *p* = 0.03).

**Conclusion:**

Monochorionic twin pregnancies conceived *via* assisted reproductive technology have a heightened risk of cesarean section and neonatal deaths. However, there is a need for further studies to supplement current evidence.

**Systematic Review Registration:** https://www.crd.york.ac.uk/prospero/display_record.php?RecordID=325133, identifier: CRD42022325133.

## Introduction

Infertility is the primary reproductive disorder affecting a large population around the globe (1). Nevertheless, the use of assisted reproductive technology (ART) like in-vitro fertilization (IVF) and intra-cytoplasmic sperm injections (ICSI) has revolutionized the management of the disease (2). Approximately 200,000 neonates are born by ART every year and data from Europe suggests that around 5,919,320 ART cycles were carried out from 1997 to 2011 (3, 4). Trends indicate that the use of ART is steadily increasing and a further larger number of procedures are expected to be performed in the near future (5). Alongside the increased use of ART, there has been a simultaneous increase in the rates of twin pregnancies (6). Indeed, it is common for clinicians to transfer more than one embryo during ART to ensure higher pregnancy rates (6). Despite improved outcomes reported by single embryo transfer, the number of multiple pregnancies with ART is still high with assisted conception resulting in approximately 20% multiple pregnancies (3, 7).

It has been well-documented that twin pregnancies have worse maternal and neonatal outcomes as compared to singleton pregnancies (8). Furthermore, several meta-analysis studies have also established that singleton pregnancies resulting from assisted conception have poor outcomes as compared to those conceived spontaneously (9, 10). ART results in an increased risk of cesarean sections, hypertensive disorders of pregnancy (HDP), gestational diabetes, preterm rupture of membranes, and preterm delivery. Also, babies born after ART have an increased risk of congenital anomalies, perinatal mortality, being small for gestational age (SGA), and having low birth weight (10). In this context, it is important to also compare outcomes of twin pregnancies conceived with ART vs. those conceived spontaneously. In a recent meta-analysis study, Qin et al. (11) pooled data from 15 cohort studies and compared outcomes of dichorionic twin pregnancies conceived with and without ART. They reported an increased risk of placenta previa, cesarean section, preterm birth, low birth weight, and congenital anomalies with assisted conception as compared to spontaneous conception. While their review focused only on dichorionic twin pregnancies, literature on the outcomes of monochorionic twin pregnancies with ART is ever scarce. This can be partially attributed to the low occurrence of monochorionic twin pregnancies with ART compared to spontaneous conception (around 2% vs. 22% respectively) (11, 12). Previously, many studies have compared outcomes of monochorionic twin pregnancies conceived with and without ART but no systematic review has been conducted to pool the available evidence. Considering the rarity of monochorionic twin pregnancies with ART, such pooled evidence can provide high-quality data for effective counseling of women carrying monochorionic twins after ART. Therefore, we carried out the present review to assess whether monochorionic twin pregnancies conceived by assisted conception have worse maternal and neonatal outcomes than those conceived naturally.

## Materials and methods

The PROSPERO registration no of the review is CRD42022325133. The review is reported based on the PRISMA statement (13).

### Literature search

Two reviewers electronically searched PubMed, ScienceDirect, CENTRAL, Embase, and Google Scholar databases up to 14 April 2022. We utilized both free-text and MeSH keywords for the literature search. The keywords utilized were “assisted reproductive technology”, “ART”, “assisted conception”, “assisted reproduction”, “in-vitro fertilization”, “IVF”, “intra-cytoplasmic injections”, “ICSI”, “artificial insemination”, “intrauterine insemination”, “spontaneous conception”, “pregnancy outcome” and “complications” in various combinations. The search queries common to all databases are shown in Supplementary Table S1. Following the database search, we electronically deduplicated the results. Initial search results were screened by article titles and abstracts. Studies found to be relevant were identified and downloaded and cross-examined against the eligibility criteria. The entire exercise was conducted by two reviewers independently. Differences between the reviewers, if any, were resolved by consensus. We read the reference list of included studies to look if any other studies were missed. If any study was not retrievable or in case of missing data, the corresponding authors were contacted by e-mail.

### Eligibility criteria

We framed the following inclusion criteria to select studies for the review: (1) All types of English-language studies conducted on women with monochorionic twin pregnancies. (2) Studies comparing outcomes of twin pregnancies with assisted conception vs. those with spontaneous conception. There was no restriction placed on the type of assisted conception. (3) Studies with a minimal sample size of 10 pregnancies per group. (4) Studies reporting any pregnancy or neonatal outcomes.

We excluded studies with duplicate data. If we found two studies with duplicate data, the study including larger number of patients and maximum data was included.

### Data extraction

The following data were extracted from the studies: author details, study type, study database, sample size, type of assisted conception, maternal age, parity, and outcomes. We initially extracted all maternal and neonatal outcome data from the included studies. However, only those outcomes reported by at least three studies were selected for quantitative analysis. Based on the availability of data the following outcomes were selected for this meta-analysis: HDP, cesarean section, twin-twin transfusion syndrome (TTTS), very premature delivery (defined as <32 weeks), birth weight, intra-uterine death, neonatal death, and SGA babies.

### Quality assessment

As all included studies were observational, we used the Newcastle-Ottawa scale (NOS) to assess study quality (14). NOS has three main domains, namely, selection of study population, comparability, and outcomes and these are awarded a maximum of four, two, and three points respectively. Study quality was examined by two authors independently and any differences were solved by consensus.

### Statistical analysis

We used the software “Review Manager” [RevMan, version 5.3; Nordic Cochrane Centre (Cochrane Collaboration), Copenhagen, Denmark; 2014] for the quantitative analysis using the inverse-variance method. Ordinal data were pooled using odds ratios (OR) with 95% confidence intervals (CI) while continuous data were pooled using mean difference (MD) and 95% CI in a random effects model. The DerSimonian and Laird model was used for the meta-analysis.

We analyzed inter-study heterogeneity using the *I*^2^ statistic. *I*^2^ scores of 25%–50% denoted low, while values of 50%–75% and >75% represented medium and substantial heterogeneity respectively. Since <10 studies were available for the review, we did not use funnel plots to assess publication bias.

## Results

**Figure 1:**
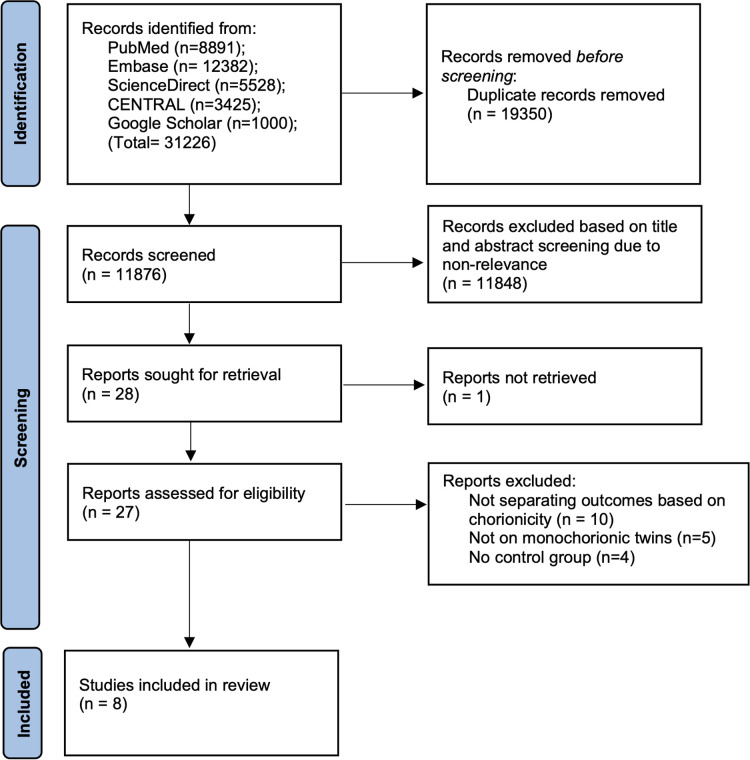
Study flow chart.

Following the complete database search we found 11,876 unique articles (Figure 1). These were then meticulously screened to retrieve 28 records relevant to the review. 27 articles could be extracted for full-text analysis. 19 were excluded and eight studies were identified for inclusion in this review (15–22).

The studies were published between 2006 and 2020 (Table 1). All were retrospective observational studies except for one prospective study. The studies were conducted in various countries around the globe but the majority of studies were on the European population (15, 16, 19–22). Four of the studies (15–17, 22) included patients undergoing IVF and ICSI in the assisted conception group. A total of 3,048 pregnancies were included in the eight studies (15–22). The included studies compared a total of 337 assisted conception monochorionic twin pregnancies with 2,711 spontaneously conceived monochorionic twin pregnancies. Further baseline characteristics of the study participants were not universally reported by the included studies. The NOS score of the majority of studies was 7 (16, 18–22). Only two studies (15, 17) were of high quality with a score of 9.

**Table 1:** Details of included studies.

Study	Study type	Location	Database	Types of AC	Groups	Sample size (n)	Maternal age (Mean ± SD)	Nulliparous (%)	Outcomes included in the review	NOS
Couck 2020 (15)	R	Belgium	University Hospitals Leuven	IVF, ICSI	AC SC	80 574	32 ± 4 30 ± 4	NR	C-section, TTTS, very premature delivery, birth weight, IUD, neonatal death	9
Hack 2018 (16)	R	Netherlands	University Medical Center Utrecht	IVF, ICSI	AC SC	29 223	33.7 ± 3.1 31.4 ± 4.8	79.3 42.7	HDP, C-section, very premature delivery, birth weight, IUD, neonatal death, SGA	7
Sun 2016 (17)	R	China	Shanghai First Maternity and Infant Hospital	IVF, ICSI	AC SC	29 427	32.9 ± 3.5 29.2 ± 3.9	90 83.1	HDP, very premature delivery, IUD, neonatal death, SGA	9
Bregar 2016 (20)	R	Slovenia	Slovenian National Perinatal Information System	NR	AC SC	45 438	32.1 ± 3.7 29.4 ± 4.5	77.8 53.2	HDP, C-section, TTTS, very premature delivery, birth weight, IUD, neonatal death	7
Ben-Ami 2016 (18)	R	Multinational	Multicentric	IVF	AC SC	43 284	33.8 ± 5.5 31.6 ± 5.4	NR	TTTS	7
Simoes 2015 (19)	R	Portugal	Maternity Hospital Dr. Alfredo da Costa	All types	AC SC	25 483	33.9 ± 5.4 29.9 ± 5.3	84 53.6	HDP, C-section, TTTS, very premature delivery, IUD, neonatal death	7
Ghalili 2013 (21)	R	Australia	Royal Prince Alfred Hospital	IVF	AC SC	76 218	36.9 ± NR 33	NR	C-section, TTTS, very premature delivery, birth weight, IUD, SGA	7
Sperling 2006 (22)	P	Denmark, Sweden	Multicentric	IVF, ICSI	AC SC	10 64	31 ± 7 35 ± 4	NR	Birth weight, IUD, neonatal death	7

IVF, in-vitro fertilization; ICSI, intra-cytoplasmic sperm injections; AC, assisted conception; SC, spontaneous conception; NR, not reported; NOS, Newcastle Ottawa scale; P, prospective; R retrospective; SGA, small for gestational age; TTTS, twin-twin transfusion syndrome; IUD, intra-uterine death.

### Meta-analysis

**Figure 2:**
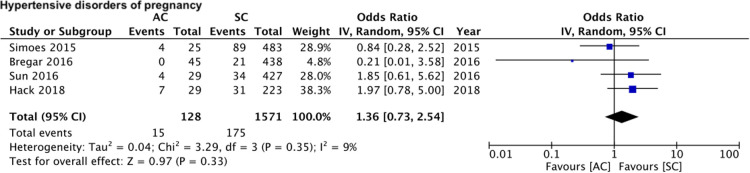
Meta-analysis of HDP between assisted and spontaneously conceived monochorionic twin pregnancies.

**Figure 3:**
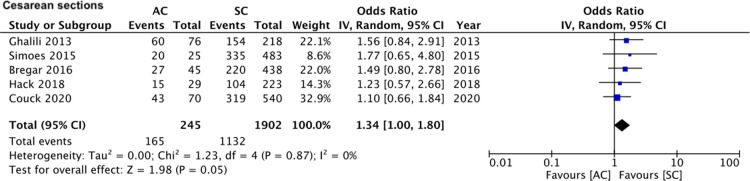
Meta-analysis of cesarean sections between assisted and spontaneously conceived monochorionic twin pregnancies.

**Figure 4:**
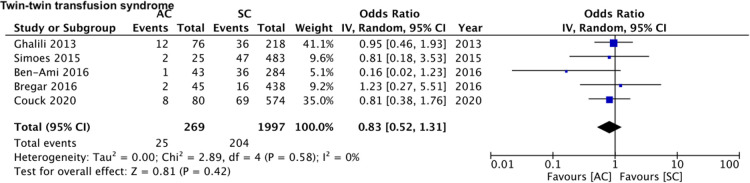
Meta-analysis of TTTS between assisted and spontaneously conceived monochorionic twin pregnancies.

**Figure 5:**
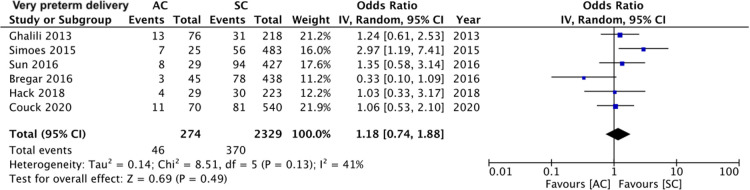
Meta-analysis of very preterm delivery between assisted and spontaneously conceived monochorionic twin pregnancies.

A total of four studies (16, 17, 19, 20) reported data on HDP (128 assisted conceptions vs. 1,571 spontaneous conceptions). Meta-analysis revealed that the mode of the conception of monochorionic twin pregnancies had no impact on the risk of HDP (OR: 1.36 95% CI, 0.73, 2.54 *I*^2^ = 9% overall effect *p* = 0.03) (Figure 2). Comparing data of 245 assisted conceptions with 1,902 spontaneous conception from five studies (15, 16, 19–21), we noted a marginally increased risk of cesarean sections in the assisted conception sub-group (OR: 1.34 95% CI, 1.00, 1.80 *I*^2^ = 0% overall effect *p* = 0.05) (Figure 3). The risk of TTTS was reported in five studies (15, 18–21) comparing 269 assisted conceptions with 1,997 spontaneous conceptions. Pooled analysis showed no difference in the risk of TTTS between the assisted and spontaneous conception groups (OR: 0.83 95% CI, 0.52, 1.31 *I*^2^ = 0% overall effect *p* = 0.42) (Figure 4). The incidence of very preterm delivery was reported by six studies (15–17, 19–21). Comparing data of 274 assisted conceptions with 2,329 spontaneous conception, there was no difference in the risk of very preterm delivery between the two groups (OR: 1.18 95% CI, 0.74, 1.88 *I*^2^ = 41% overall effect *p* = 0.49) (Figure 5).

**Figure 6:**
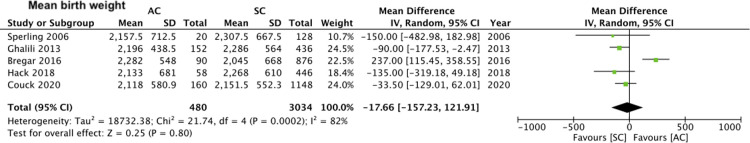
Meta-analysis of mean birth weight between assisted and spontaneously conceived monochorionic twin pregnancies.

**Figure 7:**
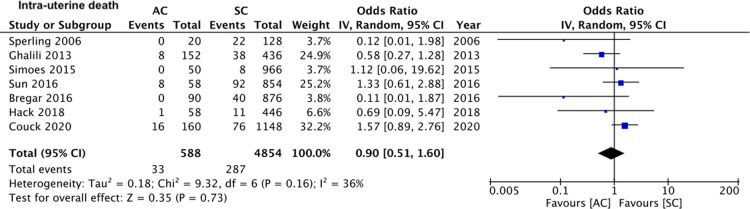
Meta-analysis of intra-uterine death between assisted and spontaneously conceived monochorionic twin pregnancies.

**Figure 8:**
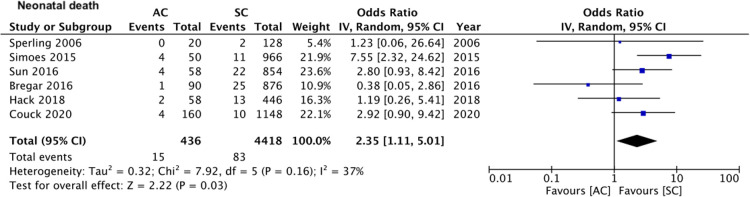
Meta-analysis of neonatal death between assisted and spontaneously conceived monochorionic twin pregnancies.

**Figure 9:**
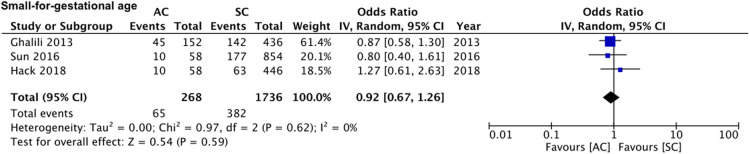
Meta-analysis of SGA between assisted and spontaneously conceived monochorionic twin pregnancies.

On pooled analysis of data from five studies (15, 16, 20–22) comparing 480 assisted conceptions with 3,034 spontaneous conceptions, we noted no statistically significant difference in the mean birth weights of all neonates between assisted and spontaneous conception groups (MD: −17.66 95% CI, −157.23, 121.91 *I*^2^ = 82% overall effect *p* = 0.80) (Figure 6). Comparing data of 588 assisted conceptions with 5,854 spontaneous conceptions from seven studies (15–17, 19–22), we noted that the mode of conception had no impact on the risk of intra-uterine death (OR: 0.90 95% CI, 0.51, 1.60 *I*^2^ = 36% overall effect *p* = 0.73) (Figure 7). However, pooled analysis of data from six studies (15–17, 19, 20, 22) comparing 436 assisted conceptions with 4,418 spontaneous conceptions did denote increased risk of neonatal death with assisted conceptions as compared to spontaneous conceptions (OR: 2.35 95% CI, 1.11, 5.01 *I*^2^ = 37% overall effect *p* = 0.03) (Figure 8). Data on the incidence of SGA was available only from three studies (16, 17, 21) comparing 268 assisted conceptions with 1,736 spontaneous conceptions. Meta-analysis indicated no difference in the risk of SGA babies between the two groups (OR: 0.92 95% CI, 0.67, 1.26 *I*^2^ = 0% overall effect *p* = 0.59) (Figure 9).

## Discussion

The results of our meta-analysis indicate that that monochorionic twin pregnancies conceived by ART have an increased risk of cesarean section and neonatal deaths. However, there seems to be no difference in the risk of HDP, TTTS, very preterm delivery, mean birth weight, intra-uterine death, and SGA between ART conceived vs. spontaneously conceived monochorionic twins.

Over the last 30-years, there has been a 100 times increase in the incidence of multiple gestations, which now constitute approximately 3% of all live births in the USA (23). This significant increase in numbers is primarily due to the increased use of ART in clinical practice. While the number of dizygotic twins has remarkably increased with assisted conception, research suggests that ART also increased the incidence of monozygotic splitting from 1 in 250 in spontaneous conceptions to around 1 in 50 (15, 24). Knopman et al. (24) in their study of 6,223 gestations have noted a 2.1% incidence of monozygotic splitting with ART with factors like young oocyte age, treatment year, and extended culture (or embryo stage at transfer) conferring the highest risk. Since ART significantly increases the rate of monochorionic twinning and such twins are already at an increased risk of complications due to factors like the presence of vascular anastomoses and potential transfusion imbalances (25), it is imperative to understand if assisted conception further worsens outcomes in such cases. To the best of our knowledge, this is the first systematic effort in literature to collate evidence on the outcomes of monochorionic twin pregnancies conceived by assisted vs. spontaneous conception.

Amongst the maternal outcomes, we noted no difference in the risk of HDP but a marginally increased risk of cesarean sections in twins conceived *via* assisted conception. Important to note is that in the meta-analysis on risk of cesarean sections, none of the included studies noted a higher risk with assisted conception in their respective cohorts, and it was only in the overall pooled analysis which demonstrated a marginal increase in the risk of cesarean section (the lower end of the 95% CI was 1). This could be due to the small sample size of the included studies which could include only a limited number of monochorionic twin pregnancies in the assisted conception group. Therefore, it is plausible that the small sample size of the individual studies could not discern such differences which were revealed by our pooled analysis. The results of our study are somewhat coherent with the outcomes of dichorionic twin pregnancies conceived by assisted vs. spontaneous conception. In the meta-analysis by Qin et al. (11), there was no difference in the risk of HDP, gestational diabetes mellitus, placental abruption, premature rupture of membranes, and postpartum hemorrhage between the two groups. However, the risk of placenta previa and cesarean sections was significantly increased with assisted conception. A meta-analysis study has also found an increased risk of HDP, cesarean section, gestational diabetes, premature rupture of membranes, and antepartum hemorrhage in singleton pregnancies conceived by ART as compared to those conceived spontaneously (10). However, as mentioned earlier, several important outcomes like the risk of placenta previa, premature rupture of membranes, and post-partum hemorrhage outcomes could not be compared in our meta-analysis due to a lack of data. It needs to be mentioned that only Sun et al. (17) have compared these outcomes and have noted a significantly increased risk of placenta previa and post-partum hemorrhage in ART conceived monochorionic twin pregnancies. Further studies are therefore needed to supplement the current data on maternal outcomes.

The existence of placental anastomosis in monochorionic twin pregnancies leads to some peculiar complications like TTTS. The incidence of TTTS in monochorionic twins is around 10%–15% and it is usually seen between 16–26 weeks of gestation (25, 26). The occurrence of a minimum of one unidirectional arteriovenous anastomosis is needed for the development of this phenomenon. TTTS could result in progressive sequelae of hypovolemia, oliguria, and oligohydramnios in the donor and hypervolemia, polyuria, and polyhydramnios in the recipient; and it is recognized as an important cause of fetal death and anomalies in monochorionic pregnancies (27). In our analysis, we noted no difference in the risk of TTTS based on the mode of conception. The incidence was 9.3% in the assisted conception group and 10.2% in the spontaneous conception group. Indeed, Ben-Ami et al. (18) have suggested that the lower incidence of TTTS in ART-assisted twins may be due to a different biological process that lies at the core of IVF conception of monozygotic twinning. The lack of difference in TTTS also coincided with no difference in the risk of intra-uterine death between the two groups. It is known that TTTS is the most common cause of mortality in monochorionic twin pregnancies and the lower incidence of this phenomenon may have a confounding effect on mortality rates between assisted and spontaneously conceived monochorionic twin pregnancies (18).

Amongst other neonatal outcomes, we noted no difference in the risk of very premature birth, mean birth weights, and SGA between the two groups. Qin et al. (11) in the meta-analysis comparing outcomes of dichorionic twin pregnancies with assisted and spontaneous conceptions have also reported no difference in the risk of very low birth weight, SGA, intrauterine growth restrictions, neonatal intensive care unit admissions, and neonatal respiratory distress syndrome between the two groups. However, they did note a significantly higher risk of very premature birth and congenital malformations in the assisted conception group. In our study, we did note a statistically significant two-fold increase in the risk of neonatal mortality with assisted conceptions. Nevertheless, it should be noted that these are crude mortality rates and several confounders can affect neonatal mortality. Few of the factors are very premature birth and congenital anomalies like found in the study of Qin et al. (11) for dichorionic twin pregnancies. While our limited data did not find any difference in very premature birth between the two groups, data on congenital anomalies was not available. Future studies incorporating these factors could provide better evidence. Furthermore, it is possible that other than the mode of conception, baseline differences in subfertile women undergoing ART and fertile women conceiving naturally could have influenced neonatal mortality. Future studies must take into account factors like maternal smoking, hypertension, gestational diabetes, body mass index, etc. while assessing the difference in the risk of mortality.

It needs to be mentioned that the lack of consistent reporting amongst the included studies is an important limitation of our review. There are several maternal and neonatal outcomes that are important in clinical practice but were not reported in the included studies. At the protocol stage, it was planned to include all those outcomes which were reported by at least three studies to have sufficient data for quantitative analysis. Hence, the current review should not be considered exhaustive as it provides only selective evidence on the outcomes of monochorionic twin pregnancies conceived by assisted vs. spontaneous conception.

Other limitations of our review are as follows. Firstly, only a the small number of studies could be included in our review. All of them were retrospective in nature and of limited sample size. Such studies are prone to bias and therefore the results should be interpreted with caution. Furthermore, due to the limited number of studies in each meta-analysis, we could not explore the cause of inter-study heterogeneity by a meta-regression or subgroup analysis. Secondly, not all studies were population-based studies including all pregnancies from the first trimester onwards. Referral of only complicated cases to tertiary centers is a possibility and this could have led to skewed outcomes. Thirdly, we were unable to different outcomes based on the type of ART due to a lack of data. Research suggests that frozen embryo transfer may result in better perinatal outcomes in terms of birth weight and spontaneous second-trimester miscarriages as compared to fresh embryo transfer (28). Fourthly, we were also unable to differentiate outcomes based on amnionicity of the gestations due to inadequate data in the included studies. At this point it is unclear if monochorionic monoamniotic and monochorionic diamniotic pregnancies have different outcomes based on method of conception. Fifthly, all outcomes were derived from cohorts that were not matched at baseline. The role of confounding variables influencing the outcomes cannot be negated. Lastly, we included only English language studies in our review and this may have led to publication bias.

Nevertheless, there are important strengths to our review. This is the first meta-analysis study on an important topic. Secondly, by pooling data from several studies of small sample sizes, our results present the largest comparison of monochorionic twin pregnancies conceived by ART and spontaneously. These results would therefore act as a preliminary guide while managing and counseling women with monochorionic pregnancies. The low heterogeneity in the majority of outcomes is an added advantage.

## Conclusion

Pooled analysis of a limited number of studies suggests that monochorionic twin pregnancies conceived by ART have an increased risk of cesarean section and neonatal deaths. There seems to be no difference in the risk of HDP, TTTS, very preterm delivery, mean birth weight, intra-uterine death, and SGA between ART conceived vs. spontaneously conceived monochorionic twins. There is a need for further research taking into account confounding factors and other important maternal and neonatal outcomes to strengthen current evidence.

## Data Availability

The original contributions presented in the study are included in the article/Supplementary Material, further inquiries can be directed to the corresponding author/s.

## References

[1] Chiware TM Vermeulen N Blondeel K Farquharson R Kiarie J Lundin K IVF and other ART in low- and middle-income countries: a systematic landscape analysis. Hum Reprod Update. (2021) 27:213–28. 10.1093/HUMUPD/DMAA047 33238297 PMC7903111

[2] Sharma RS Saxena R Singh R. Infertility / assisted reproduction: a historical / modern scientific perspective. Indian J Med Res. (2018) 148:10–4. 10.4103/IJMR.IJMR_636_18 PMC6469376 30964077

[3] De Mouzon J Lancaster P Nygren KG Sullivan E Zegers-Hochschild F Mansour R World collaborative report on assisted reproductive technology, 2002. Hum Reprod. (2009) 24:2310–20. 10.1093/HUMREP/DEP098 19474459

[4] Ferraretti AP Nygren K Andersen AN de Mouzon J Kupka M Calhaz-Jorge C Trends over 15 years in ART in Europe: an analysis of 6 million cycles. Hum Reprod Open. (2017) 2017:1–10. 10.1093/HROPEN/HOX012 PMC6276702 31486803

[5] Kushnir VA Barad DH Albertini DF Darmon SK Gleicher N. Systematic review of worldwide trends in assisted reproductive technology 2004–2013. Reprod Biol Endocrinol. (2017) 15:1–9. 10.1186/S12958-016-0225-2/FIGURES/3 28069012 PMC5223447

[6] Sunderam S Kissin DM Zhang Y Jewett A Boulet SL Warner L Assisted reproductive technology surveillance—United States, 2017. MMWR Surveill Summ. (2020) 69:1–24. 10.15585/MMWR.SS6909A1 PMC7755269 33332294

[7] Christian DG. Single embryo transfer in all infertile couples treated with assisted reproduction produces excellent results and avoids multiple births. Swiss Med Wkly. (2021) 151. 10.4414/SMW.2021.20499 34000057

[8] Meyer R Orvieto R Israel A Mohr-Sasson A Timerman Y Gorodesky T Outcomes of singleton versus twin pregnancies in the fifth and sixth decades. Eur J Obstet Gynecol Reprod Biol. (2018) 231:255–61. 10.1016/J.EJOGRB.2018.11.007 30445376

[9] Qin JB Sheng XQ Wu D Gao SY You YP Yang TB Worldwide prevalence of adverse pregnancy outcomes among singleton pregnancies after in vitro fertilization/intracytoplasmic sperm injection: a systematic review and meta-analysis. Arch Gynecol Obstet. (2017) 295:285–301. 10.1007/S00404-016-4250-3 27896474

[10] Pandey S Shetty A Hamilton M Bhattacharya S Maheshwari A. Obstetric and perinatal outcomes in singleton pregnancies resulting from IVF/ICSI: a systematic review and meta-analysis. Hum Reprod Update. (2012) 18:485–503. 10.1093/HUMUPD/DMS018 22611174

[11] Qin JB Wang H Sheng X Xie Q Gao S. Assisted reproductive technology and risk of adverse obstetric outcomes in dichorionic twin pregnancies: a systematic review and meta-analysis. Fertil Steril. (2016) 105:1180–92. 10.1016/J.FERTNSTERT.2015.12.131 26801066

[12] Carter EB Bishop KC Goetzinger KR Tuuli MG Cahill AG. The impact of chorionicity on maternal pregnancy outcomes. Am J Obstet Gynecol. (2015) 213:390.e1–e7. 10.1016/J.AJOG.2015.05.027 25986034

[13] Page MJ McKenzie JE Bossuyt PM Boutron I Hoffmann TC Mulrow CD The PRISMA 2020 statement: an updated guideline for reporting systematic reviews. Int J Surg. (2021) 88. 10.1016/j.ijsu.2021.105906 33789826

[14] Wells G Shea B O’Connell D Peterson J Welch V Losos M The Newcastle-Ottawa Scale (NOS) for assessing the quality of nonrandomised studies in meta-analyses. Available from: http://www.ohri.ca/programs/clinical_epidemiology/oxford.asp (Accessed October 30, 2020).

[15] Couck I Van Nylen L Deprest J Lewi L. Monochorionic twins after in-vitro fertilization: do they have poorer outcomes? Ultrasound Obstet Gynecol. (2020) 56:831–6. 10.1002/UOG.21973 31909558

[16] Hack KEA Vereycken MEMS Torrance HL Koopman-Esseboom C Derks JB. Perinatal outcome of monochorionic and dichorionic twins after spontaneous and assisted conception: a retrospective cohort study. Acta Obstet Gynecol Scand. (2018) 97:717–26. 10.1111/AOGS.13323 29430623 PMC5969062

[17] Sun L Zou G Wei X Chen Y Zhang J Okun N Clinical outcomes after assisted reproductive technology in twin pregnancies: chorionicity-based comparison. Sci Rep. (2016) 6:26869. 10.1038/SREP26869 PMC4886640 27243373

[18] Ben-Ami I Molina FS Battino S Daniel-Spiegel E Melcer Y Flöck A Monochorionic diamniotic in vitro fertilization twins have a decreased incidence of twin-to-twin transfusion syndrome. Fertil Steril. (2016) 105:729–33. 10.1016/J.FERTNSTERT.2015.11.036 26690011

[19] Simões T Queirós A Marujo AT Valdoleiros S Silva P Blickstein I. Outcome of monochorionic twins conceived by assisted reproduction. Fertil Steril. (2015) 104:629–32. 10.1016/J.FERTNSTERT.2015.06.002 26093266

[20] Trojner Bregar A Blickstein I Verdenik I Lucovnik M Tul N. Outcome of monochorionic-biamniotic twins conceived by assisted reproduction: a population-based study. J Perinat Med. (2016) 44:881–5. 10.1515/JPM-2015-0406 27219096

[21] Ghalili A McLennan A Pedersen L Kesby G Hyett J. Outcomes of monochorionic diamniotic twin pregnancies: a comparison of assisted and spontaneous conceptions. Aust N Z J Obstet Gynaecol. (2013) 53:437–42. 10.1111/AJO.12105 23829294

[22] Sperling L Kiil C Larsen LU Qvist I Schwartz M Jørgensen C Naturally conceived twins with monochorionic placentation have the highest risk of fetal loss. Ultrasound Obstet Gynecol. (2006) 28:644–52. 10.1002/UOG.3820 17001739

[23] Chauhan SP Scardo JA Hayes E Abuhamad AZ Berghella V. Twins: prevalence, problems, and preterm births. Am J Obstet Gynecol. (2010) 203:305–15. 10.1016/J.AJOG.2010.04.031 20728073

[24] Knopman JM Krey LC Oh C Lee J McCaffrey C Noyes N. What makes them split? Identifying risk factors that lead to monozygotic twins after in vitro fertilization. Fertil Steril. (2014) 102:82–9. 10.1016/J.FERTNSTERT.2014.03.039 24794318

[25] Lewi L Jani J Blickstein I Huber A Gucciardo L Van Mieghem T The outcome of monochorionic diamniotic twin gestations in the era of invasive fetal therapy: a prospective cohort study. Am J Obstet Gynecol. (2008) 199:514.e1–e8. 10.1016/J.AJOG.2008.03.050 18533114

[26] Lewi L Deprest J Hecher K. The vascular anastomoses in monochorionic twin pregnancies and their clinical consequences. Am J Obstet Gynecol. (2013) 208:19–30. 10.1016/J.AJOG.2012.09.025 23103301

[27] Gratacós E Ortiz JU Martinez JM. A systematic approach to the differential diagnosis and management of the complications of monochorionic twin pregnancies. Fetal Diagn Ther. (2012) 32:145–55. 10.1159/000342751 23006773

[28] Shavit M Miller N Schreiber H Asali A Ravid D Harlev A Twin pregnancies and perinatal outcomes: a comparison between fresh and frozen embryo transfer: a two-centre study. Reprod Biomed Online. (2019) 38:241–8. 10.1016/J.RBMO.2018.11.004 30579823

