# Supplementary material for: Comparison of outcomes of monochorionic twin pregnancies conceived by assisted reproductive technology vs. spontaneous conceptions: A systematic review and meta-analysis
Source: Front Pediatr. 2022 Oct 13;10:962190. doi: 10.3389/fped.2022.962190 (PMC9608745; doi:10.3389/fped.2022.962190)
Supplement: Supplementary file 1 [file Table1.docx]

Supplementary Table 1: Search strategy

| **Search number** | **Query** | **Search Details** |
| --- | --- | --- |
| **1** | (((assisted reproductive technology) OR (ART)) AND (spontaneous conception)) AND (pregnancy outcome) | ("reproductive techniques, assisted"[MeSH Terms] OR ("reproductive"[All Fields] AND "techniques"[All Fields] AND "assisted"[All Fields]) OR "assisted reproductive techniques"[All Fields] OR ("assisted"[All Fields] AND "reproductive"[All Fields] AND "technology"[All Fields]) OR "assisted reproductive technology"[All Fields] OR ("art"[MeSH Terms] OR "art"[All Fields])) AND (("spontaneous"[All Fields] OR "spontaneously"[All Fields]) AND ("conceptional"[All Fields] OR "conceptive"[All Fields] OR "fertilization"[MeSH Terms] OR "fertilization"[All Fields] OR "conception"[All Fields] OR "conceptions"[All Fields])) AND ("pregnancy outcome"[MeSH Terms] OR ("pregnancy"[All Fields] AND "outcome"[All Fields]) OR "pregnancy outcome"[All Fields]) |
| **2** | (((assisted reproductive technology) OR (ART)) AND (spontaneous conception)) AND (complications) | ("reproductive techniques, assisted"[MeSH Terms] OR ("reproductive"[All Fields] AND "techniques"[All Fields] AND "assisted"[All Fields]) OR "assisted reproductive techniques"[All Fields] OR ("assisted"[All Fields] AND "reproductive"[All Fields] AND "technology"[All Fields]) OR "assisted reproductive technology"[All Fields] OR ("art"[MeSH Terms] OR "art"[All Fields])) AND (("spontaneous"[All Fields] OR "spontaneously"[All Fields]) AND ("conceptional"[All Fields] OR "conceptive"[All Fields] OR "fertilization"[MeSH Terms] OR "fertilization"[All Fields] OR "conception"[All Fields] OR "conceptions"[All Fields])) AND ("complicances"[All Fields] OR "complicate"[All Fields] OR "complicated"[All Fields] OR "complicates"[All Fields] OR "complicating"[All Fields] OR "complication"[All Fields] OR "complication s"[All Fields] OR "complications"[MeSH Subheading] OR "complications"[All Fields]) |
| **3** | (((assisted conception) OR (assisted reproduction)) AND (spontaneous conception)) AND (pregnancy outcome) | ((("assistances"[All Fields] OR "assistant s"[All Fields] OR "assistants"[All Fields] OR "assisted"[All Fields] OR "assisting"[All Fields] OR "assistive"[All Fields] OR "dental assistants"[MeSH Terms] OR ("dental"[All Fields] AND "assistants"[All Fields]) OR "dental assistants"[All Fields] OR "assistant"[All Fields] OR "helping behavior"[MeSH Terms] OR ("helping"[All Fields] AND "behavior"[All Fields]) OR "helping behavior"[All Fields] OR "assist"[All Fields] OR "assistance"[All Fields] OR "assists"[All Fields]) AND ("conceptional"[All Fields] OR "conceptive"[All Fields] OR "fertilization"[MeSH Terms] OR "fertilization"[All Fields] OR "conception"[All Fields] OR "conceptions"[All Fields])) OR (("assistances"[All Fields] OR "assistant s"[All Fields] OR "assistants"[All Fields] OR "assisted"[All Fields] OR "assisting"[All Fields] OR "assistive"[All Fields] OR "dental assistants"[MeSH Terms] OR ("dental"[All Fields] AND "assistants"[All Fields]) OR "dental assistants"[All Fields] OR "assistant"[All Fields] OR "helping behavior"[MeSH Terms] OR ("helping"[All Fields] AND "behavior"[All Fields]) OR "helping behavior"[All Fields] OR "assist"[All Fields] OR "assistance"[All Fields] OR "assists"[All Fields]) AND ("reproduction"[MeSH Terms] OR "reproduction"[All Fields] OR "reproductions"[All Fields] OR "reproductive"[All Fields] OR "reproductively"[All Fields] OR "reproductives"[All Fields] OR "reproductivity"[All Fields]))) AND (("spontaneous"[All Fields] OR "spontaneously"[All Fields]) AND ("conceptional"[All Fields] OR "conceptive"[All Fields] OR "fertilization"[MeSH Terms] OR "fertilization"[All Fields] OR "conception"[All Fields] OR "conceptions"[All Fields])) AND ("pregnancy outcome"[MeSH Terms] OR ("pregnancy"[All Fields] AND "outcome"[All Fields]) OR "pregnancy outcome"[All Fields]) |
| **4** | (((assisted conception) OR (assisted reproduction)) AND (spontaneous conception)) AND (complications) | ((("assistances"[All Fields] OR "assistant s"[All Fields] OR "assistants"[All Fields] OR "assisted"[All Fields] OR "assisting"[All Fields] OR "assistive"[All Fields] OR "dental assistants"[MeSH Terms] OR ("dental"[All Fields] AND "assistants"[All Fields]) OR "dental assistants"[All Fields] OR "assistant"[All Fields] OR "helping behavior"[MeSH Terms] OR ("helping"[All Fields] AND "behavior"[All Fields]) OR "helping behavior"[All Fields] OR "assist"[All Fields] OR "assistance"[All Fields] OR "assists"[All Fields]) AND ("conceptional"[All Fields] OR "conceptive"[All Fields] OR "fertilization"[MeSH Terms] OR "fertilization"[All Fields] OR "conception"[All Fields] OR "conceptions"[All Fields])) OR (("assistances"[All Fields] OR "assistant s"[All Fields] OR "assistants"[All Fields] OR "assisted"[All Fields] OR "assisting"[All Fields] OR "assistive"[All Fields] OR "dental assistants"[MeSH Terms] OR ("dental"[All Fields] AND "assistants"[All Fields]) OR "dental assistants"[All Fields] OR "assistant"[All Fields] OR "helping behavior"[MeSH Terms] OR ("helping"[All Fields] AND "behavior"[All Fields]) OR "helping behavior"[All Fields] OR "assist"[All Fields] OR "assistance"[All Fields] OR "assists"[All Fields]) AND ("reproduction"[MeSH Terms] OR "reproduction"[All Fields] OR "reproductions"[All Fields] OR "reproductive"[All Fields] OR "reproductively"[All Fields] OR "reproductives"[All Fields] OR "reproductivity"[All Fields]))) AND (("spontaneous"[All Fields] OR "spontaneously"[All Fields]) AND ("conceptional"[All Fields] OR "conceptive"[All Fields] OR "fertilization"[MeSH Terms] OR "fertilization"[All Fields] OR "conception"[All Fields] OR "conceptions"[All Fields])) AND ("complicances"[All Fields] OR "complicate"[All Fields] OR "complicated"[All Fields] OR "complicates"[All Fields] OR "complicating"[All Fields] OR "complication"[All Fields] OR "complication s"[All Fields] OR "complications"[MeSH Subheading] OR "complications"[All Fields]) |
| **5** | (((In-vitro fertilization) OR (IVF)) AND (spontaneous conception)) AND (pregnancy outcome) | ("in vitro fertilisation"[All Fields] OR "fertilization in vitro"[MeSH Terms] OR ("fertilization"[All Fields] AND "vitro"[All Fields]) OR "fertilization in vitro"[All Fields] OR ("vitro"[All Fields] AND "fertilization"[All Fields]) OR "in vitro fertilization"[All Fields] OR ("j in vitro fert embryo transf"[Journal] OR "ivf"[All Fields])) AND (("spontaneous"[All Fields] OR "spontaneously"[All Fields]) AND ("conceptional"[All Fields] OR "conceptive"[All Fields] OR "fertilization"[MeSH Terms] OR "fertilization"[All Fields] OR "conception"[All Fields] OR "conceptions"[All Fields])) AND ("pregnancy outcome"[MeSH Terms] OR ("pregnancy"[All Fields] AND "outcome"[All Fields]) OR "pregnancy outcome"[All Fields]) |
| **6** | (((In-vitro fertilization) OR (IVF)) AND (spontaneous conception)) AND (complications) | ("in vitro fertilisation"[All Fields] OR "fertilization in vitro"[MeSH Terms] OR ("fertilization"[All Fields] AND "vitro"[All Fields]) OR "fertilization in vitro"[All Fields] OR ("vitro"[All Fields] AND "fertilization"[All Fields]) OR "in vitro fertilization"[All Fields] OR ("j in vitro fert embryo transf"[Journal] OR "ivf"[All Fields])) AND (("spontaneous"[All Fields] OR "spontaneously"[All Fields]) AND ("conceptional"[All Fields] OR "conceptive"[All Fields] OR "fertilization"[MeSH Terms] OR "fertilization"[All Fields] OR "conception"[All Fields] OR "conceptions"[All Fields])) AND ("complicances"[All Fields] OR "complicate"[All Fields] OR "complicated"[All Fields] OR "complicates"[All Fields] OR "complicating"[All Fields] OR "complication"[All Fields] OR "complication s"[All Fields] OR "complications"[MeSH Subheading] OR "complications"[All Fields]) |
| **7** | (((intra-cytoplasmic sperm injection) OR (ICSI)) AND (spontaneous conception)) AND (pregnancy outcome) | (("intra-cytoplasmic"[All Fields] AND ("sperm s"[All Fields] OR "spermatozoa"[MeSH Terms] OR "spermatozoa"[All Fields] OR "sperm"[All Fields] OR "sperms"[All Fields]) AND ("inject"[All Fields] OR "injectability"[All Fields] OR "injectant"[All Fields] OR "injectants"[All Fields] OR "injectate"[All Fields] OR "injectates"[All Fields] OR "injected"[All Fields] OR "injectible"[All Fields] OR "injectibles"[All Fields] OR "injecting"[All Fields] OR "injections"[MeSH Terms] OR "injections"[All Fields] OR "injectable"[All Fields] OR "injectables"[All Fields] OR "injection"[All Fields] OR "injects"[All Fields])) OR ("sperm injections, intracytoplasmic"[MeSH Terms] OR ("sperm"[All Fields] AND "injections"[All Fields] AND "intracytoplasmic"[All Fields]) OR "intracytoplasmic sperm injections"[All Fields] OR "icsi"[All Fields])) AND (("spontaneous"[All Fields] OR "spontaneously"[All Fields]) AND ("conceptional"[All Fields] OR "conceptive"[All Fields] OR "fertilization"[MeSH Terms] OR "fertilization"[All Fields] OR "conception"[All Fields] OR "conceptions"[All Fields])) AND ("pregnancy outcome"[MeSH Terms] OR ("pregnancy"[All Fields] AND "outcome"[All Fields]) OR "pregnancy outcome"[All Fields]) |
| **8** | (((intra-cytoplasmic sperm injection) OR (ICSI)) AND (spontaneous conception)) AND (complications) | (("intra-cytoplasmic"[All Fields] AND ("sperm s"[All Fields] OR "spermatozoa"[MeSH Terms] OR "spermatozoa"[All Fields] OR "sperm"[All Fields] OR "sperms"[All Fields]) AND ("inject"[All Fields] OR "injectability"[All Fields] OR "injectant"[All Fields] OR "injectants"[All Fields] OR "injectate"[All Fields] OR "injectates"[All Fields] OR "injected"[All Fields] OR "injectible"[All Fields] OR "injectibles"[All Fields] OR "injecting"[All Fields] OR "injections"[MeSH Terms] OR "injections"[All Fields] OR "injectable"[All Fields] OR "injectables"[All Fields] OR "injection"[All Fields] OR "injects"[All Fields])) OR ("sperm injections, intracytoplasmic"[MeSH Terms] OR ("sperm"[All Fields] AND "injections"[All Fields] AND "intracytoplasmic"[All Fields]) OR "intracytoplasmic sperm injections"[All Fields] OR "icsi"[All Fields])) AND (("spontaneous"[All Fields] OR "spontaneously"[All Fields]) AND ("conceptional"[All Fields] OR "conceptive"[All Fields] OR "fertilization"[MeSH Terms] OR "fertilization"[All Fields] OR "conception"[All Fields] OR "conceptions"[All Fields])) AND ("complicances"[All Fields] OR "complicate"[All Fields] OR "complicated"[All Fields] OR "complicates"[All Fields] OR "complicating"[All Fields] OR "complication"[All Fields] OR "complication s"[All Fields] OR "complications"[MeSH Subheading] OR "complications"[All Fields]) |
| **9** | (((artificial insemination) OR (intra-uterine insemination)) AND (spontaneous conception)) AND (pregnancy outcome) | ("insemination, artificial"[MeSH Terms] OR ("insemination"[All Fields] AND "artificial"[All Fields]) OR "artificial insemination"[All Fields] OR ("artificial"[All Fields] AND "insemination"[All Fields]) OR ("intra-uterine"[All Fields] AND ("inseminate"[All Fields] OR "inseminated"[All Fields] OR "inseminates"[All Fields] OR "inseminating"[All Fields] OR "insemination"[MeSH Terms] OR "insemination"[All Fields] OR "inseminations"[All Fields] OR "inseminator"[All Fields] OR "inseminators"[All Fields]))) AND (("spontaneous"[All Fields] OR "spontaneously"[All Fields]) AND ("conceptional"[All Fields] OR "conceptive"[All Fields] OR "fertilization"[MeSH Terms] OR "fertilization"[All Fields] OR "conception"[All Fields] OR "conceptions"[All Fields])) AND ("pregnancy outcome"[MeSH Terms] OR ("pregnancy"[All Fields] AND "outcome"[All Fields]) OR "pregnancy outcome"[All Fields]) |
| **10** | (((artificial insemination) OR (intra-uterine insemination)) AND (spontaneous conception)) AND (complications) | ("insemination, artificial"[MeSH Terms] OR ("insemination"[All Fields] AND "artificial"[All Fields]) OR "artificial insemination"[All Fields] OR ("artificial"[All Fields] AND "insemination"[All Fields]) OR ("intra-uterine"[All Fields] AND ("inseminate"[All Fields] OR "inseminated"[All Fields] OR "inseminates"[All Fields] OR "inseminating"[All Fields] OR "insemination"[MeSH Terms] OR "insemination"[All Fields] OR "inseminations"[All Fields] OR "inseminator"[All Fields] OR "inseminators"[All Fields]))) AND (("spontaneous"[All Fields] OR "spontaneously"[All Fields]) AND ("conceptional"[All Fields] OR "conceptive"[All Fields] OR "fertilization"[MeSH Terms] OR "fertilization"[All Fields] OR "conception"[All Fields] OR "conceptions"[All Fields])) AND ("complicances"[All Fields] OR "complicate"[All Fields] OR "complicated"[All Fields] OR "complicates"[All Fields] OR "complicating"[All Fields] OR "complication"[All Fields] OR "complication s"[All Fields] OR "complications"[MeSH Subheading] OR "complications"[All Fields]) |
